# Potential immunomodulatory effects of latent toxoplasmosis in humans

**DOI:** 10.1186/1471-2334-11-274

**Published:** 2011-10-18

**Authors:** Jaroslav Flegr, Ilja Stříž

**Affiliations:** 1 Faculty of Science, Biology Departments, Charles University in Prague, Prague, Czech Republic; 2 Institute of Clinical & Experimental Medicine, Department for Clinical and Transplant Immunology, Prague, Czech Republic

## Abstract

**Background:**

About 30% of the population worldwide are infected with the protozoan parasite *Toxoplasma gondii*. Latent toxoplasmosis has many specific behavioral and physiological effects on the human organism. Modified reactivity of the immune system has been suggested to play a key role in many of these effects. For example, the immunosuppression hypothesis explains the higher probability of the birth of male offspring observed in *Toxoplasma*-positive humans and mice by the protection of the (more immunogenic) male embryos against abortion.

**Methods:**

Here we searched for indices of immunosuppression in *Toxoplasma*-positive subjects by comparing clinical records of immunology outpatients.

**Results:**

Our cohort study showed that the male patients with latent toxoplasmosis had decreased and the *Toxoplasma*-positive women had increased leukocyte, NK-cell and monocyte counts in comparison with controls. The B-cell counts were reduced in both *Toxoplasma*-positive men and women. The difference between *Toxoplasma*-positive and *Toxoplasma*-negative subjects diminished with the decline of the specific *Toxoplasma *antibody titre (a proxy for the length of infection), which is consistent with the observed decreasing strength of the effect of latent toxoplasmosis on human reproduction. The prevalence of toxoplasmosis in 128 male patients was unusually low (10.9%) which contrasted with normal prevalence in 312 female patients (23.7%) and in general population Prague (20-30%).

**Conclusions:**

Latent toxoplasmosis has immunomodulatory effects in human and probably protects men against some classes of immunopathological diseases. The main limitation of the present study was the absence of the data on the immunoreactivity of immune cells subpopulations. Therefore further studies are needed to search for indices of immunosuppression in human using more specific markers.

## Background

*Toxoplasma gondii*, a parasitic protozoan related to *Plasmodium*, infects about 30% of the human population worldwide. Latent toxoplasmosis, characterized by the life-long presence of cysts of the parasite in different host tissues, including the nervous system, and by the presence of anamnestic *Toxoplasma *IgG antibodies in the serum, was long considered asymptomatic. In the past 20 years, several effects of this form of parasitosis on the human organism were described in the literature. For example, latent toxoplasmosis increases the risk of schizophrenia [1] and Parkinson's disease [2] influences human personality and behavior [3,4], impairs psychomotor performance, enhances the risk of suicide [5], of traffic accident [6-9] and increases probability of the birth of male offspring [10,11]. Reportedly, the activity of the immune system is likely to play an important role in many of the observed effects of *Toxoplasma *infection. For example, the impairment of the immune system has been suggested to be at least partly responsible for the observed association between toxoplasmosis and schizophrenia [3]. Also, many of the observed behavioural effects of toxoplasmosis might be a result of the increased level of dopamine in the brain tissue in response to IL-2 produced by immune cells in the sites of local inflammation in the infected brain [12-14]. Similarly, the effect of latent toxoplasmosis on human reproduction, not only on the probability of the birth of male offspring, but also on the probability of the birth of a child with Down syndrome [15] and on the length of pregnancy [16], has been assumed to be a consequence of toxoplasmosis-associated immunosuppression. It is well known that most of the embryos, especially the more immunogenic male embryos and those with various chromosomal aberrations and physical malformations, are aborted in early phases of pregnancy[17,18]. The immunosuppression hypothesis suggests that *Toxoplasma *relaxes the stringency of some mechanisms of quality control of early embryos to increase the probability of its transmission to the next generation through the congenitally infected offspring [10,11].

Many reports are available concerning the effect of acute toxoplasmosis on the immunity of humans or mice [19-21]. However, the data showing similar effects in congenital toxoplasmosis are absent. The results obtained in infected laboratory female mice showed that mice in the early phase of latent infection exhibited temporarily increased production of IL-12 and decreased production of IL-10. In accordance with the immunosuppression hypothesis, the mice showed decreased production of IL-2 and nitric oxide and decreased synthesis of DNA in the mixed lymphocyte assay in the early and also in the late phases of latent toxoplasmosis [22]. It is difficult to study such effects in mouse models, as the duration of acute and post-acute stages of infection approaches the normal length of life in this species. On the other hand, there is a striking difference between the about one-month acute infection and life-long latent infection in humans. In the present cohort study, we searched for indices of immunomodulation in humans with latent toxoplasmosis by comparing the available clinical records, namely the flow cytometry and haematological data, in *Toxoplasma*-positive (infected) and *Toxoplasma*-negative (noninfected) outpatients undergoing routine immunological tests at the Institute of Clinical and Experimental Medicine in Prague.

## Methods

### Subjects

The experimental design of present study was a prospective cohort study. Clinical data of all immunology outpatients of the Institute of Clinical and Experimental Medicine in Prague from 2008-2009 were anonymized and analyzed for possible association between latent toxoplasmosis (anamnestic titres of *Toxoplasma *antibodies in frozen samples of sera collected for clinical analysis) and immune cell counts. Diagnosis (ICD-10) of most subjects (250 ×) was D89.9 (disorder involving the immune mechanism, unspecified) and J30.1-3 seasonal allergic rhinitis (117 ×). Other diagnoses observed in our analysed population were A69.2 (2 ×), B009 (3 ×), B007 (2 ×), D80.0 (1 ×), D80.2 (1 ×), D80.6 (1 ×), D81.9 (10 ×), D83.9 (2 ×), D89.8, E06.3 (2 ×), F48.0 (3 ×), H10.1 (1 ×), H10.9 (1 ×), J01.0 (1 ×), J06.8 (1 ×), J06.9 (1 ×), J30.0 (1 ×), J30.3 (12 ×), J45.0 (11 ×), J45.9 (10 ×), K30 (1 ×), K50.9 (6 ×), M05.9 (1 ×), M35.0 (1 ×), N76.1 (2 ×), O26.9 (1 ×), T63.4 (1 ×), T78.4 (1 ×). We analysed the whole population of patients and also two subpopulations separately: patients with immunodeficiencies D89.9 and patients with allergies, J30.0-4, J45.0, J45.9. The subjects with D89.1 diagnosis were mostly untreated, while the standard medication with second generation antihistamines and topical corticosteroids was used in allergic patients. This treatment can influence (decrease) the cell counts but does not affect proportion of particular cell types in the peripheral blood. There were no patients under systemic corticotherapy in any of the groups. The study was approved by the Institutional Review Board of the Faculty of Science, Charles University (Apr. No. 2008/3).

### Serological tests for toxoplasmosis

All serological tests were carried out in the National Reference Laboratory for Toxoplasmosis, National Institute of Public Health, Prague. All study subjects were screened for specific *Toxoplasma *IgG antibodies and those with high IgG levels were tested for IgM antibodies by ELISA (IgG: SEVAC, Prague, IgM: TestLine, Brno; optimized for early detection of acute toxoplasmosis) and the complement fixation test (CFT) (SEVAC, Prague) [23], at dilutions between 1:4 and 1:1024. The subjects IgM negative (positivity index < 0.9) and IgG positive by ELISA (positivity index > 1.0) were considered latent toxoplasmosis positive. None of the study subjects had a CFT titre higher than 1:128 or an index for IgM higher than 0.9.

### Statistics

The effects of sex and age on the risk of latent toxoplasmosis were tested with logistic regression. Effects of toxoplasmosis, sex and age on the immune cells counts were quantified with GLM and correlation between CFT titres and immune cells counts in subjects with latent toxoplasmosis was analyzed with a non-parametric test, namely partial Kendall regression analysis with age as a covariate. All variables except the fraction of lymphocytes and erythrocytes were log transformed to achieve an approximately normal distribution.

## Results

The studied population consisted of 440 patients, 312 women and 128 men. The mean age of 238 *Toxoplasma*-negative women (40.22, SD = 17.21) was significantly lower than that of 74 (23.7%) *Toxoplasma*-positive women (49.11, SD = 13.72), *P *< 0.0001. Similarly, 114 *Toxoplasma*-negative men (36.04, SD = 18.28) were significantly younger than 14 (10.9%) *Toxoplasma*-positive men (50.14, SD = 19.32), *P *< 0.008. The logistic regression with the dependent variable toxoplasmosis and independent variables sex and age showed that men had a significantly lower probability of *Toxoplasma *infection than women (*P *= 0.009, OR = 0.43, CI_95 _= 0.23-0.81) and the probability of being *Toxoplasma *positive increased with each year of age (*P *< 0.0001, OR = 1.035, CI_95 _= 1.02-1.05). No effect of the diagnosis on probability of the *Toxoplasma *infection was observed when the binary variable diagnosis (immunodeficiency yes/no) was included into the model (*P *= 0.779, OR = 0.920, CI_95 _= 0.51-1.65).

General linear model analyses with particular haematological or flow-cytometry parameters as dependent factors, toxoplasmosis (binary) and sex (binary) as independent factors and age (continuous) as a confounding variable showed that toxoplasmosis correlates with several haematological and cytometric parameters, namely the leukocyte, CD19, CD16 + 56 and monocyte counts, mostly shifting the size of immune cell subpopulations in opposite directions in men and women (Tables 1 and 2). The separate GLM analyses performed for patients with immunodeficiencies and patients with allergies and asthma showed that that the former subpopulation was probably responsible for the observed effects.

**Table 1:** Comparison of the haematological data from *Toxoplasma*-negative and *Toxoplasma*-positive men and women

	Age *P *	Sex *P *	Toxo *P *	sex-toxo *P *	women-neg mean	women-pos mean	men-neg mean	men-pos mean
*all immunology outpatients N = 440 *								

leukocytes (× 10^6^/L)	0.412	0.002*	0.502	**0.054**	6913.136	7229.730	6572.973	5750.000

neutrophils (× 10^9^/L)	0.011*	0.009*	0.811	0.184	4.059	4.471	3.729	3.396

lymphocytes (× 10^9^/L)	0.000*	0.047*	0.719	0.152	2.015	1.985	1.997	1.644

monocytes (× 10^9^/L)	0.751	0.506	0.107	0.024*	0.508	0.518	0.548	0.451

eosinophils (× 10^9^/L)	0.423	0.227	0.793	0.699	0.185	0.201	0.223	0.216

basophils (× 10^9^/L)	0.581	0.016*	0.185	0.250	0.047	0.046	0.042	0.032

*patients with allergies, seasonal allergic rhinitis J.30.1-4, N = 117 *								

leukocytes (× 10^6^/L)	0.600	0.191	0.268	0.351	689.059	774.375	667.500	668.000

neutrophils (× 10^9^/L)	0.511	0.111	0.326	0.339	4.058	4.924	3.733	3.892

lymphocytes (× 10^9^/L)	0.006*	0.961	0.666	0.991	2.020	1.953	2.128	1.990

monocytes (× 10^9^/L)	0.701	0.329	0.502	0.421	0.475	0.534	0.555	0.544

eosinophils (× 10^9^/L)	0.278	0.641	0.320	0.496	0.200	0.269	0.224	0.218

basophils (× 10^9^/L)	0.858	0.120	0.483	0.630	0.050	0.049	0.041	0.032

*patients with immunodeficiencies, D89.9, N = 250 *								

leukocytes (× 10^6^/L)	0.958	0.049*	0.152	**0.053**	670.876	690.000	669.677	535.714

neutrophils (× 10^9^/L)	**0.078**	**0.067**	0.282	0.184	4.026	4.176	3.832	3.207

lymphocytes (× 10^9^/L)	0.000*	0.129	0.256	**0.051**	1.937	1.977	2.022	1.440

monocytes (× 10^9^/L)	0.800	0.329	0.039*	0.018*	0.505	0.512	0.549	0.406

eosinophils (× 10^9^/L)	0.919	**0.065**	0.806	0.937	0.180	0.190	0.241	0.246

basophils (× 10^9^/L)	0.121	0.175	0.172	0.201	0.045	0.045	0.044	0.031

Columns 3-6 show the significance (*P *) of effects of age, sex, latent toxoplasmosis and toxoplasmosis-sex interaction for the whole population, patients with seasonal allergic rhinitis and patients with immunodeficiencies.Columns 7-10 show the arithmetic means of various variables in particular subpopulations. The *P *value = 0.000 means *P *< 0.0005, the significant *P *-values (*P *< 0.05) are denoted with asterisks, the trends *P *< 0.10 are, printed in bold.

**Table 2:** Comparison of the flow cytometry data from *Toxoplasma*-negative and *Toxoplasma*-positive men and women

	Age *P *	Sex *P *	Toxo *P *	sex-toxo *P *	women-neg mean	women-pos mean	men-neg mean	men-pos mean
*all immunology outpatients N = 440 *								

CD3 (× 10^6^/L)	0.000*	0.009*	0.599	0.164	1549.190	1507.236	1481.540	1190.571

CD19 (× 10^6^/L)	0.000*	0.002*	0.045*	0.012*	254.091	224.514	262.205	155.143

CD4 (× 10^6^/L)	0.000*	0.000*	0.474	0.149	989.026	977.625	878.469	722.786

CD8 (× 10^6^/L)	0.000*	0.395	0.974	0.439	512.725	490.028	527.973	431.429

CD16 + 56 (× 10^6^/L)	0.709	0.861	0.508	0.016*	215.268	243.722	257.795	208.857

CD4/CD8	0.001*	0.021*	0.835	0.875	2.162	2.211	1.809	2.020

*patients with allergies, seasonal allergic rhinitis and asthma J.30.1-4, N = 117 *								

CD3 (× 10^6^/L)	0.002*	0.291	0.990	0.920	1566.797	1444.375	1544.031	1331.600

CD19 (× 10^6^/L)	0.000*	0.852	0.723	0.208	223.625	232.063	279.469	205.200

CD4 (× 10^6^/L)	0.026*	0.121	0.621	0.731	979.719	930.875	855.938	839.000

CD8 (× 10^6^/L)	**0.074**	0.704	0.549	0.856	532.062	443.125	574.125	491.000

CD16 + 56 (× 10^6^/L)	0.180	0.913	0.866	0.032*	223.652	319.500	283.788	236.000

CD4/CD8	0.011*	0.727	0.648	0.400	2.069	2.174	1.661	2.222

*patients with immunodeficiencies, D89.9, N = 250 *								

CD3 (× 10^6^/L)	0.000*	**0.077**	0.423	0.112	1479.986	1509.234	1481.694	1105.000

CD19 (× 10^6^/L)	0.000*	0.141	0.116	0.210	249.216	220.213	251.613	128.143

CD4 (× 10^6^/L)	**0.065**	0.007*	0.251	**0.089**	950.075	972.426	884.903	635.000

CD8 (× 10^6^/L)	0.000*	0.829	0.877	0.299	486.552	505.149	534.807	419.429

CD16 + 56 (× 10^6^/L)	0.992	0.875	0.271	0.161	211.745	218.851	250.985	187.571

CD4/CD8	0.000*	**0.023**	0.463	0.570	2.176	2.232	1.816	1.792

Columns 3-6 show the significance (*P *) of effects of age, sex, latent toxoplasmosis and toxoplasmosis-sex interaction for the whole population, patients with seasonal allergic rhinitis and patients with immunodeficiencies. Columns 7-10 show the arithmetic means of various variables in particular subpopulations. The *P *value = 0.000 means *P *< 0.0005, the significant *P *-values (*P *< 0.05) are denoted with asterisks, the trends *P *< 0.10 are, printed in bold..

**Figure 1:**
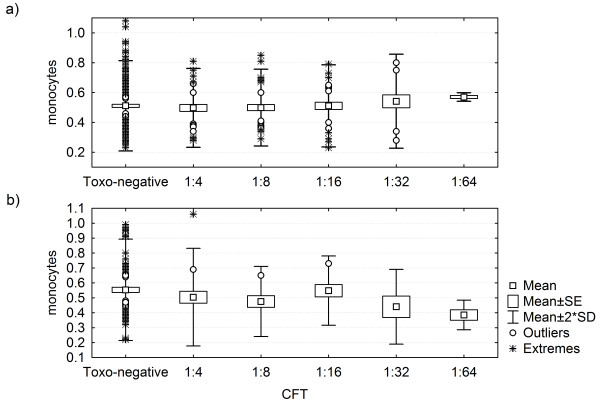
**Correlation between the CFT titres of *Toxoplasma *antibodies and monocyte counts**. The CFT titres decrease with the length of *Toxoplasma *infection. The monocyte count (y-axis) is shown in 10^9 ^per liter. a) women, b) men. Number of patients with titres 64, 32, 16, 8, 4 and <4 was 4, 14, 37, 45, 45 and 295, respectively.

Although the length of infection in particular *Toxoplasma*-positive patients is unknown, it can be estimated from the level of *Toxoplasma *antibodies measured by CFT. Unlike the irregularly fluctuating concentrations of antibodies measured by ELISA, the CFT titres have been reported to decrease slowly and relatively regularly in years after the infection [23]. Results of the partial Kendall regression analysis with age as a covariate showed that leukocytes, CD19, CD16 + 56 and monocytes had a non-significant tendency to return to normal with decreasing CFT titres of *Toxoplasma *antibodies, for monocytes in women this tendency was significant (partial Kendall Tau = 0.170, *P *= 0.028), see Figure 1.

## Discussion

The prevalence of latent toxoplasmosis was significantly higher in female (23.7%) than in male (10.9%) patients. Patients with or without anamnestic titres of *Toxoplasma *antibodies, i.e. with or without latent toxoplasmosis, differed from each others in several important parameters, namely in the counts of leukocytes, CD19 cells (B-cells), CD16 + 36 cells (natural killers) and monocytes. The number of B cells was lower in both infected men and women; however, all the other immune cell counts were higher in infected women and lower in infected men. Such shifts in the counts of particular immune cells to the opposite directions are likely to be side effects of the infection rather than a result of the biological adaptation of the parasite aimed to increase the probability of survival in the host organism by modulating its immunoreactivity.

The opposite effects of toxoplasmosis in men and women were also observed in the personality profile [3,24] and behaviour [25,26]. The key to such opposite behavioral and immunological effects in infected men and women might be the variation in the effects of *Toxoplasma *infection on the concentration of free testosterone. Reportedly, infected men had increased while infected women decreased testosterone levels in comparison with controls [27,28]. Testosterone is known to have specific effects on the behaviour as well as the immunity of animals, including humans. The increased level of testosterone is generally associated with the immunosuppression [29,30] which could explain both the decreased number of leukocytes, B-cells, NK-cells and monocytes in men and the increased size of leukocyte, NK-cell and monocyte populations in women. It must be remind, however, that experiments performed with artificially infected laboratory mice demonstrated decreased level of testosterone in both males and females. Moreover, we did not found any significant differences in the concentration of testosterone between *Toxoplasma*-positive and *Toxoplasma*-negative solders (nonpublished results). It is critically needed to search for changes of cell counts in the *Toxoplasma*-infected mice and also to search for possible correlation between concentration of testosterone and blood cell count in our patients.

The trend towards a slow return of the immune cell counts to normal suggests that the observed differences are a carry-out effect of acute infection rather than a consequence of latent toxoplasmosis. It must be remind that this trend was significant only for monocytes in women. For leukocytes, CD19 and CD16 + 56 cells, and monocytes in men the cell counts returned to normal value in subjects with oldest infection, however, the trend was nonsignificant due to large variance in cell counts and low number of infected subjects. The correlation between cell counts and duration of the infection also indicates that toxoplasmosis induces changes in the immune system rather than that a change in the immune system increases the risk of *Toxoplasma *infection. The return of the immune cell counts to normal is in accordance with the diminishing effect of latent toxoplasmosis on the sex ratio in women and female mice. The increased probability of the birth of male offspring was only observed in women with high or moderate levels of *Toxoplasma *antibodies and in female mice 2-4 months after the infection. In women with low or moderate levels of *Toxoplasma *antibodies and in female mice more than 4 months after the infection, the probability of the birth of male offspring was even lower than in uninfected controls. It was suggested that the increased sex ratio in women and mice in early phases of latent toxoplasmosis was caused by immunosuppression that protects the more immunogenic males embryos against abortion. For example, in humans, the sex ratio decreases from about 2.6 in the first weeks of pregnancy to about 1.06 at the time of delivery [17,18]. The diminishing effect of toxoplasmosis on immune cell counts is in accordance with the expected decrease of immunosuppression.

The most obvious problem of our study was related to low number of *Toxoplasma*-positive men in our population. The unbalanced design can result in false negative result - i.e. in missing some effects of toxoplasmosis. It must be stressed, however, that it cannot lead in false positive results, i.e. in detection of non-existent effects of toxoplasmosis.

The main limitation of the present study was the absence of the data on the immunoreactivity of immune cells subpopulations. We searched for indices of immunosuppression using only the clinical data that are routinely collected from patients with allergic diseases. Recent results obtained in infected laboratory female mice suggest that the concentrations of important interleukins are increased; however, the reactivity of immune cells measured by nitric oxide production and proliferation of stimulated spleen cells in the MLC assay are considerably decreased in comparison with uninfected controls [22]. Further studies are needed to search for indices of immunosuppression using more specific markers. The second potential limitation of the present study is the selected population group. The patients with immunodeficiences or with allergies are a rather specific cohort and their immunological data can hardly be generalized to the normal healthy population. Theoretically, subjects with toxoplasmosis-associated immunosuppression might be protected against severe allergies, thus escaping inclusion in our study. The prevalence of latent toxoplasmosis is significantly lower among our male than female patients, although in the Czech general population, the prevalence rates of toxoplasmosis are approximately the same in men and women [31]. Possibly, the *Toxoplasma*-positive men (with decreased counts of leukocytes, B-cells, NK cells and monocytes) are protected against allergies and therefore were not enrolled in our study, while the women (with increased counts of total leukocytes, NK-cells and monocytes) have higher probability of allergies and immunopathological diseases. Decreased frequency of *Toxoplasma*-positive subjects in patients with atopic disease has already reported in several studies [32-34]. It must be remind, however, the low frequency of *Toxoplasma *infected men as well as the statistically significant associations between the *Toxoplasma *infection and the cell counts were observed mostly in subjects with immunodeficiencies (D.89.9) not in patients with allergies (J.30.1-4) in our study.

## Conclusions

We can conclude that the data from the outpatient immunology clinic suggest specific effects of latent toxoplasmosis on humans. The effects decreased with the length of infection and were opposite in direction in men and women, possibly as a result of the reported opposite effects of the infection on the level of testosterone in men and women. Given the high prevalence of latent toxoplasmosis, its possible immunosuppressive effects, although relatively weak in individual patients, might have a considerable impact on the health of the world population.

## Competing interests

The authors declare that they have no competing interests.

## Authors' contributions

Both authors contributed equally to data collection and analysis. All authors have read and approved the final manuscript

## Pre-publication history

The pre-publication history for this paper can be accessed here:

http://www.biomedcentral.com/1471-2334/11/274/prepub
